# Does the cost of cancer care for people in prison differ from those in the general population? Analysis of matched English cancer registry and hospital records

**DOI:** 10.1016/j.eclinm.2024.102575

**Published:** 2024-04-29

**Authors:** Rachael Maree Hunter, Jennie Huynh, Margreet Lüchtenborg, Jo Armes, Emma Plugge, Rachel M. Taylor, Renske Visser, Elizabeth A. Davies

**Affiliations:** a Applied Health Research, Institute of Epidemiology and Health Care, University College London, United Kingdom; b Cancer Epidemiology and Cancer Services Research, Centre for Cancer, Society & Public Health, Comprehensive Cancer Centre, King's College London, United Kingdom; c National Disease Registration Service, NHS England, United Kingdom; d School of Health Sciences, University of Surrey, United Kingdom; e Faculty of Medicine, University of Southampton, United Kingdom; f Centre for Nurse, Midwife and Allied Health Professional Research (CNMAR), University College London Hospitals NHS Foundation Trust, United Kingdom

**Keywords:** Prison, Cancer, Cancer diagnosis, Cancer treatment, Cost, Economics, Health inequalities, Cancer registry, Hospital episode statistics

## Abstract

**Background:**

People in prison experience poorer mental and physical health compared to their peers in the general population. The causes are multi-dimensional ranging from lifestyle factors to poorer access to healthcare. Little is known about cancer in people in prison or how the cost of their care compares to the general population.

**Methods:**

Data on people diagnosed with cancer while in English prisons were identified in National Cancer Registration dataset and linked to Hospital Episode Statistics (HES) for the years 2012–2017. General population matched patients were identified using a 1–5 ratio, based on age, gender, year of diagnosis, cancer type and disease stage. Outpatient and inpatient HES data up to six-months from diagnosis were costed using NHS Reference costs and inflated to 2017/2018 costs.

**Findings:**

879 prison and 4326 general population cancer diagnoses were identified in HES. The adjusted six-month cost of cancer care was significantly lower for people in prison (−£1216.95% confidence interval (CI) −1638 to −795), driven by fewer outpatient attendances. However, people diagnosed in prison had higher emergency care costs (£497.95% CI 375–619). Security escorts further increased the total cost of care.

**Interpretation:**

Following a cancer diagnosis, people in English prisons have significantly lower planned care costs, but higher emergency care costs and an overall higher cost due to security escorts. Further work is required to identify ways of improving cancer care for people in prisons to ensure it is equivalent to that received by the general population.

**Funding:**

National Institute for Health and Social Care Research 16/52/53.

**Research in context**

**Evidence before this study**

People in prison experience poorer physical and mental health than their peers in the general population. Most research has focused on communicable diseases, substance misuse and mental health and there is limited evidence on the physical health needs of people in prison particularly for non-communicable diseases, including cancer. A search of the literature using PubMed between the years 1980 and 2023 revealed no evidence on the cost of cancer care for the prison population.

**Added value of this study**

This study uses routine health care data to identify people diagnosed with cancer in prison, together with a matched general population cohort. It is the first study to quantify the cost of cancer care for people diagnosed in prison and compare it with that for their peers in the general population.

**Implications of all the available evidence**

Although cancer care for the English prison population cost less than the matched general population cohort, this was driven by reductions in costs for planned care. The cost of emergency care was higher for the prison population. This, alongside evidence of lower survival rates and poorer experiences of care for people in prison points to the need to improve access to cancer care for people diagnosed with cancer in prisons in order to achieve equivalence of care.

## Introduction

His Majesty's Prison and Probation Service (HMPPS), covering England and Wales, reported 81,806 people in prison in December 2022^1^ and 87,586 in September 2023,^2^ 96% of which were men.^1^ Overall, prison populations experience poorer physical and mental health compared to their peers in the general population^3^ with higher rates of mental health problems,^4^ substance misuse,^5^ a higher prevalence and incidence of blood borne viruses (BBVs) such has human immunodeficiency virus (HIV) and hepatitis^6^ and other communicable diseases including Tuberculosis (TB).^7^ Physical health care needs are also greater in prison populations, including higher rates of diabetes and cardiovascular disease compared to non-incarcerated peers of a similar age.^8^ Due to these factors, combined with poor access to health care, people in prison have higher mortality rates than their non-incarcerated peers.^3^

The prison population in England and Wales has seen an increase in the proportion of people of older age, defined as over 50 in prisons, than the general population. The population aged over 50 now comprises over 17% of the population in 2022 which has increased from 7% in 2002.^1^ All these factors continue to increase the cost of providing health care in prison.

Given the challenges associated with providing health care in prison, the National Health Service (NHS) in England took over responsibility from the Prison Service for providing and being responsible for health care in prisons in 2006. This was partially to facilitate the “equivalence of care” that should be available to people in prison, following concern over the standard of care provided.^9^ The concept of equivalence of care refers to an agreement that people in prison should have access to the same quality of care as that available to them if they were living outside prison. This expectation is also cited in United Nations and World Health Organisation documents on prison health^10^ and is part of NHS England's policy for commissioning health care in prison.^11^

Little is known though about what access to equivalent cancer care in prison would look like. There is also currently no published evidence that we are aware of regarding the cost of cancer care for patients diagnosed with cancer in prison. This evidence would be helpful in identifying inefficiencies and inequities in the system and informing policy and service planning. The aim of this study is therefore to compare the cost of NHS cancer care for patients in England diagnosed with cancer in prison with that of the cost of cancer care in the general population using routine data from Hospital Episode Statistics (HES), which contains details of all hospital admissions and outpatient appointments in NHS hospitals in England. Data within HES also forms the basis by which hospitals are paid for the care they deliver.^12^

## Methods

### Study population and data

Cancer cases that occurred between 2012 and 2017 were identified from the comprehensive and quality assured NHS cancer registration records made by the National Cancer Registration Dataset (NCRD).^13^ The NCRD has been found to reliably and accurately capture data on all people living in England diagnosed with malignant and pre-malignant neoplasms, which includes people in prison. The cancer cases identified from the NCRD for this analysis were first primary invasive cancer in people aged between 18 and 120 with a known, self-reported gender, excluding non-melanoma skin cancer (International Classification of Diseases (ICD)-10 C44) and diagnoses of cervical cancer in situ (ICD-10 D06). Diagnoses made in prisons were identified based on publicly available prison postcodes, and time periods in which they were active.^14^ Previous work in the area has found this is a reliable way to identify people who are diagnosed with cancer whilst in prison.^15^

**Fig. 1:**
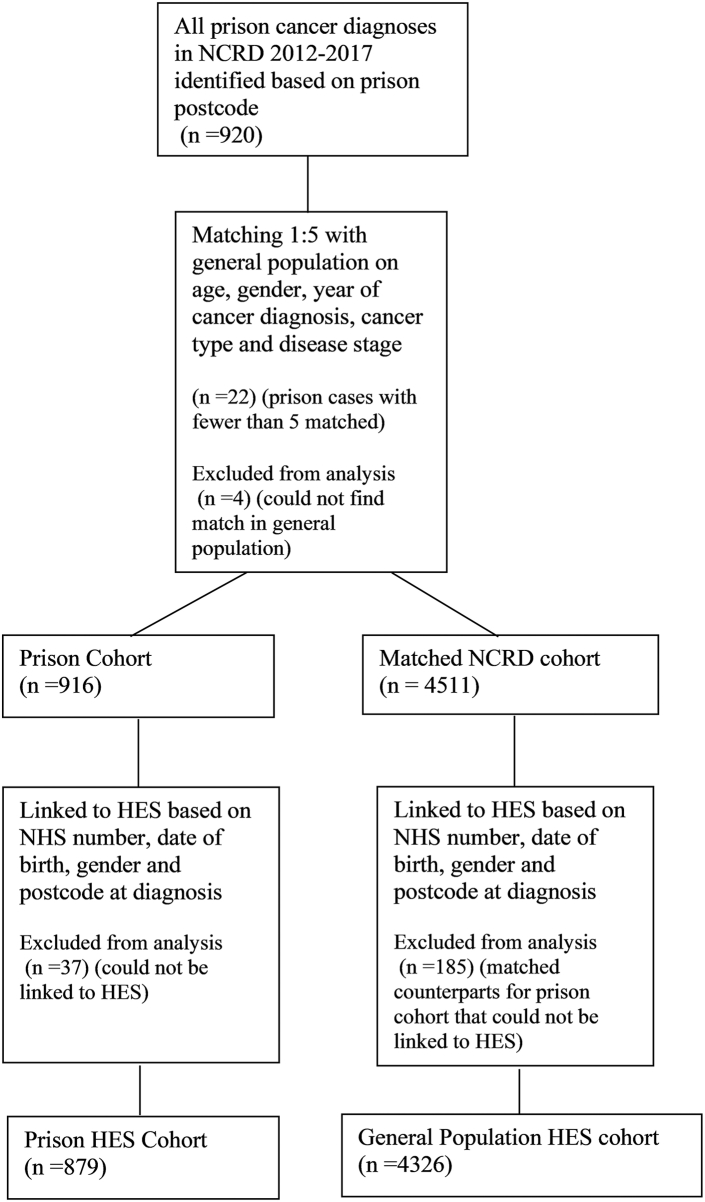
**Flow diagram of patients identified in NCRD and HES**. HES, Hospital Episode Statistics; NCRD, National Cancer Registration Dataset; NHS, National Health Service.

All patients diagnosed in prison were included in the analysis. A matched sample of patients diagnosed in the general population was randomly selected from the cancer registry at a 1:5 ratio matched on five-year age groups, self-reported gender, year of diagnosis, cancer type (3-digit ICD-10 code) and disease stage. No matching cancer patients from the general population could be identified for four prison diagnoses, and these were excluded from further analysis. All cohort members were linked at patient level to NHS hospital episode statistics (HES) based on a matching algorithm that included NHS number, date of birth, gender and postcode at diagnosis. HES data included inpatient and outpatient data only, which has been found to accurately capture the cost of cancer care compared to medical records.^16^^,^^17^ Thirty-seven prison patients could not be linked and were excluded from analysis, along with their matched cohort counterparts. The final numbers for analysis of length of stay and costs are 879 patients diagnosed in prison and 4326 general population cancer patients (see Fig. 1).

Accident and Emergency (A&E) data was not included given that the prison population do not access hospital via A&E and the challenges of identifying cancer specific A&E care in the community.

### Length of stay

Any hospital admissions in the time from 31 days before until 183 days after the date of diagnosis with a matching cancer diagnosis were used to calculate total number of bed days.

### Costs

We worked with HES resource use covering the month prior and first six-months after diagnosis, which is likely to cover all initial treatment and care related to the index cancer.

Unit costs were obtained from NHS Reference Costs 2010/11–2018/19^18^ and applied to healthcare resource groups (HRG) reported in the sample, using mean national costs for the most recent costing year that the HRG is available for. Unit costs for inpatient stays were converted to an average cost per bed-day to capture the cost impact of differences in length of stay. These were applied to inpatient bed days for emergency versus elective (non-emergency) costs. The relevant unit costs were also applied to day cases and regular day or night admissions. For HRGs that ceased to exist prior to 2018/19 costs were inflated to 18/19 costs based on HSIC.^19^ Outpatient attendances were costed using 2018/2019 unit costs, based on service code and assuming a consultant led service.

In 2009 the costs of escorts and bed-watches were transferred to the NHS, making the NHS responsible for covering the cost of a prison officer escorting a prisoner to hospital attendances, with some exceptions such as extended lengths of stay. Escort and bed watches for prison patients were costed as £168 per hospital attendance and £2232 per bed day, respectively. This is a weighted estimate based on the average time per appointment and the number of prison officers from a 2006 study of escorts and bed watches^20^ uplifted to 2018/2019 costs using the Services Producer Price Index.^21^ The costs of escorts and bed watches may be an overestimate given that we do not know if people were released and in the community during the six months from diagnosis, as well as the approximately 5% of the prison estate that are eligible for Releases on Temporary License (ROTL) and hence can apply to attend appointments without an escort.

### Statistical analysis

Descriptive statistics were calculated for the proportion of patients that used each resource use type. Means and standard deviations were calculated for attendances and bed days for patients that used that resource only. Conditional logistic regression was used to calculate the odds ratio of patients in prison accessing the resource compared to patients in the general population. Chi-square test was used to test for any differences between the two cohorts in regards to demographic characteristics.

As the distribution of costs was unlikely to be normal, we prespecified testing cost differences between prison and general population patients using generalised linear models (GLMs). The suitability of using a log link was tested for using the Stata postestimation command for the link test, which is in the form suggested by Pregibon.^22^ The family (normal, Poisson, negative binomial or gamma) was chosen based on Akaike Information Criterion (AIC).^23^ We tested versions of the model that included Charlson Score and ethnicity, but rejected including these due to poor data quality and model fit as assessed by the AIC. Residuals were plotted as a kernel density estimate alongside a normal curve to evaluate their distribution and the suitability of the models.

All odds ratios and GLMs were adjusted for matching factors (age groups, gender, year of diagnosis, and disease stage). A separate category was created for patients missing disease stage to ensure they were not omitted from the analysis. Adjusted costs and cost differences were obtained using margins with standard errors calculated using the delta method.

Analyses were conducted in Stata v17.^24^

### Sensitivity analysis

The main analysis includes patients with unknown NHS numbers. It is possible that these patients were incorrectly linked and hence they were excluded in a sensitivity analysis.

### Ethics approval

The NDRS has approval from the Confidentiality Advisory Group of the National Health Service Health Research Authority (HRA) to carry out surveillance using the data they collect on all cancer patients under section 251 of the NHS Act 2006. All analyses of national data were undertaken by researchers contracted to work within NDRS and hence ethical approval was not required for this work.

### Role of the funding source

The funder approved the study approach but had no role in study design, data collection, data analysis, data interpretation, or writing of the report.

## Results

Demographic characteristics and stage of cancer for the prison and general population cancer cohorts are reported in Table 1. Cancers in men in prison were predominately prostate (17%), lung (15%), colon and rectal (10%), testis (7%) and bladder (4%) cancers. For women in prison the majority were cervical cancer in situ (76%), with cervix and breast cancer accounting for 5% of cancers each. Further information on cancer diagnoses in the two cohorts is reported in our companion paper.^14^ People diagnosed with cancer in prison were less likely to have outpatient appointments (OR 0.31 95% CI 0.25–0.39 P < 0.0001) and planned inpatient attendances (OR 0.75 95% CI 0.64–0.88 P < 0.0001) than the matched cohort diagnosed in the general population (see Table 2). For all those who had any outpatient appointment, 20.8% (n = 275) of people diagnosed in prison had a “did not attend” recorded for an outpatient appointment compared to 13.0% (n = 448) for the matched general population cohort. This represents a significantly higher likelihood of a “did not attend” for people diagnosed in prison (OR 1.79 95% CI 1.51–2.11 P < 0.0001).

**Table 1:** Demographic characteristics for HES analysis dataset for patients diagnosed in prison compared to matched general population cohort.

	Place of diagnosis				P-value^a^
	General population		Prison		
	#	%	#	%	
**Patients**	4326		879		
**Gender**					0.936
Men	4082	94	830	94	
Women	244	6	49	6	
**Age category (years)**					1.00
18–20	29	1	6	1	
21–24	57	1	12	1	
25–29	109	3	26	3	
30–39	341	8	70	8	
40–49	693	16	140	16	
50–59	1078	25	216	25	
60–69	1144	26	231	26	
70–79	723	17	146	17	
80+	152	4	32	4	
**Stage**					0.998
1	1014	23	205	23	
2	534	12	108	12	
3	676	16	138	16	
4	1262	29	253	29	
Missing	840	19	175	20	
**Diagnosis year**					1.00
2012	505	12	102	12	
2013	662	15	135	15	
2014	697	16	142	16	
2015	775	18	157	18	
2016	867	20	176	20	
2017	820	19	167	19	
**Ethnicity**					<0.0001
White	3737	86	698	79	
Mixed	19	0	14	2	
Asian and Chinese	134	3	13	1	
Black	101	2	40	5	
Other	63	1	27	3	
Missing	272	6	87	10	
**Charlson comorbidity score**					0.086
0	3622	84	708	81	
1–2	518	12	124	14	
3+	186	4	47	5	

a P-values based on χ^2^-test, excluding missing or unknown categories.

**Table 2:** Resource use six -months post cancer diagnosis with means and standard deviations reported only for patients diagnosed in prison and the general population with a hospital attendance.

	General population	Prison	OR^b^ (95% CI)	P-value
	n = 4326	n = 879		
**Outpatient**				
Proportion (%)	93.39	82.25	0.31 (0.25–0.39)	P < 0.0001
Attendances^a^ (mean (SD))	11.97 (11.90)	8.86 (10.96)		
**Elective (planned) inpatient care**				
Proportion (%)	35.99	29.82	0.75 (0.64–0.88)	P < 0.0001
Attendances^a^ (mean (SD))	1.38 (0.93)	1.41 (1.02)		
Bed Days^a^ (mean (SD))	8.25 (10.78)	8.61 (11.08)		
**Day cases**				
Proportion (%)	45.28	36.86	0.69 (0.60–0.81)	P < 0.0001
Attendances^a^ (mean (SD))	4.51 (5.55)	3.63 (4.15)		
**Emergency inpatient care**				
Proportion (%)	16.10	14.79	0.90 (0.73–1.11)	P = 0.335
Attendances^a^ (mean (SD))	1.32 (0.68)	1.32 (0.72)		
Bed Days^a^ (mean (SD))	13.92 (19.03)	17.22 (21.77)		
**Any inpatient care**				
Proportion (%)	69.05	60.18	0.68 (0.58–0.79)	P < 0.0001
Attendances^a^ (mean (SD))	3.99 (5.12)	3.25 (3.76)		
Bed Days^a^ (mean (SD))	10.49 (15.19)	10.73 (15.38)		

a Participants with attendance recorded only.

b Conditional logistic regression model adjusted for age groups, gender, year of diagnosis, and disease stage.

Based on link tests and AIC, log link and negative binomial family were chosen for all GLM models to test for significant differences in costs. Six-month after diagnosis and one-month prior, secondary care costs for people diagnosed in prison are significantly lower than for the matched general population cohort (−£1216.20 95% CI −£1637.86 to −£794.55 P < 0.0001) with outpatient and planned inpatient care costing significantly less for those diagnosed in prison (see Table 3). Emergency inpatient care costed £497.25 (95% CI £375.23–£619.27 P < 0.0001) more per person diagnosed in prison compared to in the general population. When the cost of bed-watches and escorts is added (mean per person of £13,723) to the total cost of secondary care cancer treatment, treatment for people diagnosed in prison cost significantly more per patient than those in the matched general population cohort.

**Table 3:** Total cancer health care costs in 2018/2019 GBP for people diagnosed in the general population and in prison.

	General population	Prison	Mean difference^b^ (95% CI)	P-value
	n = 4326	n = 879		
**Outpatient**				
Cost^a^ (mean (SD))	1651 (1693)	1209 (1541)		
Adjusted^b^ total cost (mean (SE))	1544 (24)	981 (33)	−563 (−643 to −483)	P < 0.0001
**Elective (planned) inpatient care**				
Cost^a^ (mean (SD))	9382 (9902)	9424 (10,633)		
Adjusted^b^ total cost (mean (SE))	3414 (57)	2919 (101)	−495 (−715 to −275)	P < 0.0001
**Day cases**				
Cost^a^ (mean (SD))	1516 (1639)	1224 (1160)		
Adjusted^b^ total cost (mean (SE))	685 (11)	455 (16)	−230 (−267 to −193)	P < 0.0001
**Emergency inpatient care**				
Cost^a^ (mean (SD))	7473 (12,840)	8766 (12,729)		
Adjusted^b^ total cost (mean (SE))	1153 (21)	1650 (63)	497 (375–619)	P < 0.0001
**Total inpatient care**				
Cost^a^ (mean (SD))	7619 (11,144)	7589 (10,803)		
Adjusted^b^ total cost (mean (SE))	5234 (82)	4601 (157)	−634 (−977 to −291)	P < 0.0001
**Total health care costs (outpatient + inpatient)**				
Cost^a^ (mean (SD))	7154 (10,345)	6589 (10,345)		
Adjusted^b^ total cost (mean (SE))	6784 (106)	5568 (190)	−1216 (−1638 to −795)	P < 0.0001
**Total health care costs including escorts and bed-watches**				
Cost^a^ (mean (SD))	7154 (10,345)	20,312 (38,107)		
Adjusted^b^ total cost (mean (SE))	6784 (106)	17,085 (582)	10,301 (9145–11,456)	P < 0.0001

a Patients with attendance recorded only.

b Adjusted for age groups, gender, year of diagnosis, and disease stage: generalised linear model negative binomial with log link.

The results for the sensitivity analyses are reported in Appendix 1. If individuals with unknown NHS number are excluded from the analysis the conclusions remain the same.

## Discussion

The secondary health care cost of cancer care for people diagnosed in prison is £1216 less per patient on average than their peers diagnosed in the general population. This is predominately due to fewer attendances for planned inpatient care and outpatient appointments. The cost of emergency inpatient care for people diagnosed in prison was on average £497 greater per patient than for the matched general population cohort. This secondary health care also comes at the additional cost of escorts and bed-watches for people in prison, a cost that is covered by the NHS.

This is the only study that we are aware of which has attempted to quantify the cost of cancer care for people in prison, particularly compared to those in the general population. It is limited in its scope as it only includes secondary care contacts and does not include primary care or prison health care costs. Further work is required to understand the quantity and cost of prison health care for people diagnosed with cancer in prison. The equivalent would also need to be done for a matched cohort in the general population. We were also unable to include A&E data in this analysis and hence additional costs could have been missed for these attendances, particularly for people in the general population. The cost of “did not attend” was also excluded from the analysis due to the issues associated with estimating this, particularly the cost implications for prison compared to the general population.

The extensive use of routine data is a strength of this study. Research in prisons is notoriously difficult to conduct, and loss to follow-up is common.^25^ Using routine data hence reduces the bias inherent in prison studies. Nonetheless, people in prison can be commonly missing from routine health care datasets^26^ which are also known to contain some errors, particularly when calculating costs.^16^ These errors should have been equal across both groups, so although total costs might not be correct the difference in costs should be close to the true value. Co-morbidities have also been excluded from this analysis due to suspected under-reporting. This may have a knock-on effect of under costing of health care given that HRGs are determined by co-morbidity coding in files. This may have greater cost implications for the prison cohort than the general population given that co-morbidities are more common in the prison population.^3^

We have stated that these statistics and costs are for people diagnosed in prison. Some of those diagnosed in prison may have been released into the community during the six months following diagnosis, meaning that their contact with care may have changed. A recently published population-based linkage study in Connecticut in the United States of America (USA) found that cancer related mortality for people recently released from a correctional institution was even higher than people diagnosed while incarcerated.^27^ The authors attribute this to a lack of medical insurance for people recently released, as health care provision is the responsibility of the correctional system while a person is incarcerated in the USA, whereas on release they are unlikely to be insured and may not be eligible for Medicare. More evidence is required on whether the same effect would be seen in the UK given universal access to health care. A higher standardised all-cause mortality ratio following release from prison than the general population suggests that there are challenges with continuity of care.^28^

That some of the cancer care for the prison cohort may have occurred while people were in the community means also that the cost of escorts and bed-watches may have been overestimated and hence should be interpreted with caution. This is also true for people granted release on temporary licence who may not need a prison escort when attending hospital. These individuals currently make up 5% of the prison population^1^ so are unlikely to have a significant impact on the cost, but this means some caution is required in interpreting the results.

When looking at costs it is also important to look at outcomes, particularly as people who died earlier or who decide to stop treatment early cost less, and hence mortality needs to be factored in. In this case the research question would be to put a monetary value on the years of life and quality of life lost due to poorer cancer outcomes for people in prison compared to the general population. This was not possible in this study because we had limited outcome data other than mortality. The number of different cancer sites and potential outcomes would have made any decision modelling or estimation of outcomes prohibitive.

We have included six months of hospital attendances following a diagnosis from cancer to ensure that we only captured the health care cost of the index diagnosis. Although this may not capture all costs, with some cancers potentially taking longer than this to treat, it is likely to capture the vast majority of them. Further information on routes to diagnosis and curative treatment is reported in our accompanying paper.^14^ Patients can also have very different costs of cancer care depending on the type of cancer, with costs for breast cancer for example significantly differing between patients depending on cancer stage of diagnosis, age of the patient and tumour receptor status.^29^ To attempt to control for this we used a matched cohort of patients in the community and included covariates that are known to relate to costs in the analysis. We were unable to adjust for cancer site though in the analysis due to small patient numbers. This means that there is the potential for residual confounding, where not all confounding variables have been included in the analysis. This may result in the analysis showing a stronger association between being diagnosed in prison and costs than may be true.

The results of this study need to be taken in the context of other epidemiological and qualitative research that we conducted alongside this analysis. Most importantly the decreased cost of care, particularly planned care, needs to be considered in the context of a lower rate of treatment with curative intent and a lower survival rate compared to people diagnosed with comparable cancers in the general population.^14^ People with lived experience of cancer in prison also reported many barriers to accessing care.^30^ There was also evidence of reduced access to screening, potentially leading to people in prison being diagnosed at a later cancer stage.^14^ Our general population sample was matched on cancer stage though so this should not have influenced our results.

Very little research has previously been conducted examining the cost of health care in prison, particularly for non-communicable diseases. Previous costings and economic evaluations tend focus on the areas of mental health, substance misuse and communicable diseases. A systematic review of studies reporting the cost of health care in prisons found 26 studies in total, but only one relating to England and Wales.^31^ A recent report found also that approximately 40% of planned outpatient attendances made from prison are missed across all specialities compared to 25% in the general population.^32^ This is slightly higher, but with a similar relationship as our 20% of appointments being “did not attend” for prison compared to 13% of the community. A key reason for a high level of non-attendance by the prison population is lack of escort availability, given that two escorts need to escort each patient.^32^ That the funding for health care for people in prison is the responsibility of the wider health system in England makes the context for the delivery of cancer care in prisons potentially different to that of other countries. In the USA for example, prison health care falls within the budget for the department of corrections and hence potentially competes with other correctional priorities. Spending on cancer care in prisons though has seen a significant increase in the USA at $1.3 billion and 3% of total correctional care spending in 2016 to 4% of total spending in 2017.^33^ The same data is not available in England because these patients are indistinguishable from other NHS patients, with our study being the best evidence available.

Although cancer care for patients diagnosed in prison costs less for the secondary care component than for their peers diagnosed in the general population, this is alongside increased emergency attendances and reduced survival.^14^ It also comes at the additional cost associated with escorts and bed-watches. Further work is required to improve access to planned care following a cancer diagnosis in prison. It is important that any future evaluations of interventions to improve access to cancer care in prisons take account of the impact of costs as well as attempting to quantify the health benefits of the intervention. Equity considerations also need to be included as significant additional investments need to be made so that outcomes for people in prison begin to better reflect those in the general population.

## Contributors

RMH and ML designed the study and decided the analytic approach. JH was responsible for extracting and analysing the data which ML also had access to. All authors were involved in conceiving the study, contributed to the interpretation of the results and the writing of this manuscript.

## Data sharing statement

Data for this study is collated, owned, maintained, and quality assured by the National Cancer Registration and Analysis Service within NHS England. The authors do not own these data, and therefore are not permitted to share or provide this data other than in scientific communication format.

## Declaration of interests

RMH has received payments from Ministry of Justice and UK Health Security Agency for prison related consultancy work. JA is a member of the Executive Committee Member of British Psychosocial Oncology Society (unpaid) declares funding from NIHR Health Services and Social Care Delivery Programme, the Applied Research Collaboration Kent, Surrey and Sussex and the Strategic Priorities Fund 2019/20 Research England vis University of Surrey. EAD has received funding from Emory College, The Irish Cancer Society and Singapore Government.
