# Supplementary material for: Does the cost of cancer care for people in prison differ from those in the general population? Analysis of matched English cancer registry and hospital records
Source: eClinicalMedicine. 2024 Apr 29;72:102575. doi: 10.1016/j.eclinm.2024.102575 (PMC11247152; doi:10.1016/j.eclinm.2024.102575)
Supplement: Appendix 1 [file mmc1.docx]

Appendix 1: Cost Sensitivity analysis - unknown NHS numbers excluded (Adjusted for age groups, gender, year of diagnosis, and disease stage: generalised linear model negative binomial with log link):

|  |  | Adjusted cost Mean (SE) | Mean Difference |
| --- | --- | --- | --- |
|  |  |  |  |
| Outpatient | Comp | 1545.589 (24.90668) |  |
|  | Prison | 1008.12  (34.8716) | -537.469 (-620.429 to -454.509 |
| Elective inpatient | Comp | 3377.641  (57.19538) |  |
|  | Prison | 3023.497 (106.8898) | -354.144 (-583.577 to -124.711) |
| Day case | Comp | 672.9136 (10.96276) |  |
|  | Prison | 468.2676 (16.3743) | -204.646 (-242.45 to -166.842) |
| Emergency inpatient | Comp | 1164.324 (21.54516) |  |
|  | Prison | 1684.888 (64.70555) | 520.5636 (394.5648 to 646.5623) |
| Total inpatient | Comp | 5188.937 (82.27267) |  |
|  | Prison | 4731.648 (164.0583) | -457.289 (-813.371 to -101.207) |
| Total health care | Comp | 6739.039 (106.6988) |  |
|  | Prison | 5728.353 (198.1954) | -1010.69 (-1447.96 to -573.408) |
| Total health care ++ | Comp | 6739.017 (106.5665) |  |
|  | Prison | 17584.11 (608.5001) | 10845.09 (9638.346 to 12051.84) |

++ Includes cost of escorts and bed-watches
